# Application of ultrasound-guided inferior vena cava collapsibility measurement in volume assessment for patients undergoing single-shot spinal anesthesia in total hip arthroplasty

**DOI:** 10.1097/MD.0000000000040363

**Published:** 2024-11-08

**Authors:** Tao Yang, Chunyan Huang, Yulin Chen, Xuemin Lei

**Affiliations:** a Department of Anesthesiology, Neijiang Hospital of Traditional Chinese Medicine, Neijiang, Sichuan, China.

**Keywords:** hypotension, inferior vena cava, spinal anesthesia, ultrasound, volume responsiveness

## Abstract

To explore the value of ultrasound in volume assessment during the perioperative period of single-shot spinal anesthesia for total hip arthroplasty. A total of 100 patients undergoing elective surgery under spinal anesthesia at our hospital from January 2022 to January 2024 were selected. Transthoracic echocardiography was used to measure the diameter of the abdominal aorta (Ao) before anesthesia (T1), 10 minutes after anesthesia (T2), and 30 minutes after anesthesia (T3). The inferior vena cava collapsibility index (IVC-CI) and the ratio of IVCe to Ao (IVCe/Ao) were calculated. A volume load test was performed 10 minutes after anesthesia. Based on the increase in stroke volume (ΔSV) after the volume load test, patients were divided into a volume-responsive group (ΔSV ≥ 15%) and a volume-nonresponsive group (ΔSV < 15%). The reliability of inferior vena cava parameters in predicting hypotension after spinal anesthesia and the value in predicting volume responsiveness postanesthesia were evaluated using ROC curves. A total of 100 patients were enrolled, 18 were withdrawn, and a total of 82 patients were included. After the volume load test, the parameters of the volume response group and the volume nonresponse group were basically consistent, and the difference was not statistically significant (*P* > .05). The SV of volume reaction group was significantly higher than that of volume no reaction group (*P* < .05). The incidence of hypotension in the volume response group was higher than that in the non-volume response group (51.28% vs 11.63%, *χ*^2^ = 15.174, *P* < .01). The AUC for volume reactivity prediction using IVCe, IVCi, IVC-CI, and IVCe/Ao were 0.62, 0.71, 0.70, and 0.72, respectively. IVCi, IVC-CI, and IVCe/Ao were significant predictors of volume reactivity (*P* < .05). The AUC predicting persistent hypotension after spinal anesthesia using IVCe, IVCi, IVC-CI, and IVCe/Ao were 0.78, 0.79, 0.70, and 0.84, respectively. IVCe, IVCi, IVC-CI, and IVCe/Ao can predict volume reactivity before anesthesia. IVCi, IVC-CI, and IVCe/Ao predicted persistent hypotension in patients with spinal anesthesia after anesthesia, and IVCe/Ao showed the best predictive effect. Thus, IVCe/Ao is a reliable parameter for predicting persistent hypotension and assessing volumetric reactivity.

## 1. Introduction

Spinal anesthesia is widely used in clinical practice due to its rapid onset, reliable effect, low drug dose requirement, and minimal impact on the body. However, the incidence of hypotension following spinal anesthesia remains relatively high, ranging from 8% to 33%.^[1,2]^ Hypotension may result in symptoms such as dizziness, nausea, vomiting, and even inadequate perfusion of vital organs, potentially leading to cardiovascular collapse in severe cases.^[3]^ To mitigate the occurrence of hypotension, clinical measures often include pre-anesthesia fluid preload or rapid fluid infusion immediately after anesthesia.^[4]^ However, inappropriate fluid therapy can result in fluid overload and associated complications, such as pulmonary edema.^[5]^ Therefore, accurately assessing patients’ fluid tolerance is crucial for guiding optimal fluid management to correct hypotension and maintain hemodynamic stability.

Volume responsiveness is a key parameter used to evaluate a patient’s response to fluid loading, reflecting the heart’s ability to respond to an increase in preload.^[6]^ Assessing volume responsiveness helps guide fluid management decisions, thereby preventing both fluid overload and insufficient fluid therapy. However, direct evaluation of volume responsiveness often requires fluid challenge tests, which carry risks of invasiveness and potential fluid overload.^[7]^ Additionally, hypotension induced by anesthesia is complex; research indicates that even patients with good volume responsiveness may still experience persistent hypotension due to extensive vasodilation caused by spinal anesthesia, which cannot be alleviated by fluid administration, thus increasing the risk of fluid overload. Therefore, evaluating volume responsiveness alone is insufficient; it is also essential to assess whether blood pressure improves following fluid therapy. Consequently, finding reliable, noninvasive predictors to evaluate volume responsiveness and predict the resolution of hypotension after spinal anesthesia holds significant clinical value.

Traditional static preload indicators, such as central venous pressure and pulmonary artery wedge pressure, are inadequate for accurately predicting volume responsiveness.^[8]^ Advanced hemodynamic monitoring systems, such as PiCCO and FloTrac/Vigileo, provide valuable information, but their invasive nature limits their use in routine surgical procedures.^[9]^ In recent years, ultrasound assessment of the IVC has been used to evaluate volume status and volume responsiveness due to its noninvasive and simple nature.^[10]^ IVC parameters, such as the inferior vena cava collapsibility index (IVC-CI) and the ratio of maximum IVC diameter to abdominal aortic diameter (IVCe/Ao), have been used to predict hypotension and guide fluid management. The Ao diameter can effectively correct the influence of individual patient factors, such as body size and body surface area, on the IVC diameter.^[11]^ However, there is still controversy regarding the accuracy of these IVC parameters in evaluating volume responsiveness and predicting the resolution of hypotension after anesthesia.^[12–14]^

Patients undergoing total hip arthroplasty are mostly elderly and often have multiple comorbidities, making perioperative hemodynamic stability particularly important. Therefore, this study aims to investigate the value of ultrasound-measured IVC parameters (IVCe, IVCi, IVC-CI, and IVCe/Ao) in predicting volume responsiveness and the recovery of hypotension after spinal anesthesia, providing a reference for optimizing perioperative fluid management strategies.

## 2. Materials and methods

### 2.1. General information

This study was conducted from January 2022 to January 2024 in our hospital on patients undergoing elective unilateral total hip arthroplasty with single-needle subarachnoid anesthesia. A total of 100 patients were enrolled. The inclusion criteria were: age 26–72 years, ASA grade I–II, and BMI < 35. Exclusion criteria included patients unable to complete anesthesia for any reason, anesthesia level exceeding T6–T12, abnormal inferior vena cava, major organ dysfunction (heart, brain, and kidney), increased intra-abdominal pressure, difficulty with ultrasound measurement, and incomplete data. This study was approved by the Ethics Committee of our hospital.

### 2.2. Methods

#### 2.2.1. Method of anesthesia

The patient was fasted for 6 hours before surgery and given 200 mL of preloaded liquid 0.9% sodium chloride solution after the establishment of the preoperative venous channel. The patient was placed in a supine position, and after local disinfection, a spinal tap was performed in the spinal space at L3–4 or L4–5 using a 25G needle. 2.5 mL of 0.5% bupivacaine was injected into the lumbar subarachnoid space, while the patient’s blood pressure, heart rate, and blood oxygen saturation were monitored. After the injection is completed, the patient is assisted into a lateral position, which is held for 15 minutes to ensure that the anesthesia level is fixed.

#### 2.2.2. Ultrasound measurement methods

Ultrasound measurements were performed at the following 3 time points: T1: 5 minutes after entering the operating room; T2: 10 minutes after spinal anesthesia; T3: 30 minutes after spinal anesthesia. The diameters of the IVC and Ao were measured using transthoracic echocardiography, and the IVC-CI and the ratio of maximum IVC diameter to abdominal aortic diameter (IVCe/Ao) were calculated. The specific measurement methods are as follows:

IVC diameter: The patient was placed in a supine position, and an S5–1 probe was positioned vertically below the xiphoid process, tilted to the right to display the left hepatic lobe, the IVC, the left hepatic vein, and the entrance of the IVC into the right atrium. The maximum IVC diameter at end-expiration (IVCe) and the minimum IVC diameter at end-inspiration (IVCi) were measured, and IVC-CI was calculated as follows: IVC-CI = (IVCe ‐ IVCi)/IVCe × 100%.

Abdominal aorta diameter: The probe was placed below the xiphoid process for longitudinal scanning, and the abdominal Ao was measured 5–10 mm from the celiac trunk, followed by calculation of the IVCe/Ao ratio.

#### 2.2.3. Volume challenge test and grouping

At 10 minutes after spinal anesthesia (T2), all patients underwent a volume challenge test as per the study protocol, which involved intravenous infusion of hydroxyethyl starch at 8 mL/kg, with infusion completed within 20 minutes. Based on the change in SV following the fluid challenge, patients were divided into the volume-responsive group (ΔSV ≥ 15%) and the nonresponsive group (ΔSV < 15%). The ΔSV was calculated using the following formula: ΔSV = (SVT3 ‐ SVT2)/SVT2 × 100%.

#### 2.2.4. Observational indicators

Primary observational indicators: Changes in systolic blood pressure (SBP), diastolic blood pressure, heart rate (HR), and oxygen saturation; changes in the diameters of the IVC and Ao and their ratio (IVCe/Ao); changes in the IVC collapse index (IVC-CI); changes in stroke volume (SV) before and after fluid loading; incidence of hypotension after anesthesia: Hypotension in this study is defined as a reduction in SBP of more than 30% from the preoperative baseline value or an SBP of <90 mm Hg. If SBP < 90 mm Hg occurs, 0.05 mg of norepinephrine was administered intravenously as an initial treatment. If SBP remained below 90 mm Hg after 2 minutes, an additional 0.05 mg of norepinephrine was administered, and this process was repeated until blood pressure returned to the normal range. Additionally, if HR fell below 50 beats per minute, 0.5 mg of atropine was administered intravenously, with repeat doses as necessary to correct bradycardia. Recovery from hypotension was defined as an SBP recovery to more than 80% of the preoperative value within 30 minutes of treatment. Cases where SBP did not recover were defined as persistent hypotension.

Secondary observational indicators: Incidence of postoperative complications such as nausea, vomiting, and chills; dosage and side effects of anesthetic drugs.

### 2.3. Statistical methods

Data analysis was performed using SPSS 23.0 software. Statistical analyses included the following steps:

Descriptive statistics: Continuous variables were expressed as mean ± standard deviation (*x̅* ± *s*), while categorical variables were expressed as frequencies and percentages. General information (e.g., age, gender, BMI, and ASA classification) between the 2 groups of patients was compared using independent sample *t* tests and chi-square tests.

Analytical statistics: Hemodynamic parameters (SBP, diastolic blood pressure, HR, IVCe, IVCi, IVC-CI, IVCe/Ao, and SV) at different time points were compared between the fluid-responsive and nonresponsive groups using independent sample *t* tests and paired *t* tests. The incidence of hypotension between the 2 groups was compared using chi-square tests.

Correlation analysis: Pearson correlation coefficients or Spearman rank correlation coefficients were used to assess the relationship between IVC parameters (IVCe, IVCi, IVC-CI, and IVCe/Ao) and the incidence of hypotension.

Regression analysis: Multiple regression analysis was used to explore the independent predictive value of IVC parameters for the occurrence of hypotension, adjusting for potential confounders (e.g., age, gender, BMI, and ASA classification).

ROC curve analysis: ROC curves were used to evaluate the accuracy of IVC parameters (IVCe, IVCi, IVC-CI, and IVCe/Ao) in predicting hypotension and fluid responsiveness, with the area under the curve (AUC) as well as sensitivity and specificity being calculated.

## 3. Results

### 3.1. General data

Out of the initial 100 patients enrolled, 18 were excluded: 5 due to unclear inferior vena cava ultrasound images, 6 due to unclear cardiac ultrasound images, and 7 due to incomplete data collection. A total of 82 patients were included in the final analysis, with 39 in the fluid-responsive group and 43 in the nonresponsive group. There were no statistically significant differences between the 2 groups in terms of age, gender, BMI, ASA classification, SBP, or HR (*P* > .05), indicating comparability. See Table 1 for details.

**Table 1:** Comparison of general information between 2 groups of patients (*x̅* ± *s*).

Group	Cases	Age (years, *x̅* ± *s*)	Male/female (cases)	BMI (*x̅* ± *s*)	ASA classification (I/II, cases)	SBPT1 (mm Hg, *x̅* ± *s*)	HRT1 (times/min, *x̅* ± *s*)
Unreactive group	43	55.4 ± 11.4	22/21	23.2 ± 2.7	23/20	129.5 ± 15.9	73.7 ± 11.2
Reactive group	39	59.6 ± 9.7	21/18	23.0 ± 2.3	19/20	131.3 ± 15.9	72.6 ± 11.6
*t*/*χ*^2^ value		1.776	0.059	0.435	0.186	0.531	0.459
*P*-value		.080	.808	.665	.666	.597	.648

*Note*: SBPT1 is the SBP at the end of 5 min of entry; HRT1 is the HR at the end of 5 min of entry.

HRT1 = hear rate before anesthesia, SBPT1 = systolic blood pressure before anesthesia.

### 3.2. Comparison of parameters before and after volume loading test in both groups

**Figure 1.**
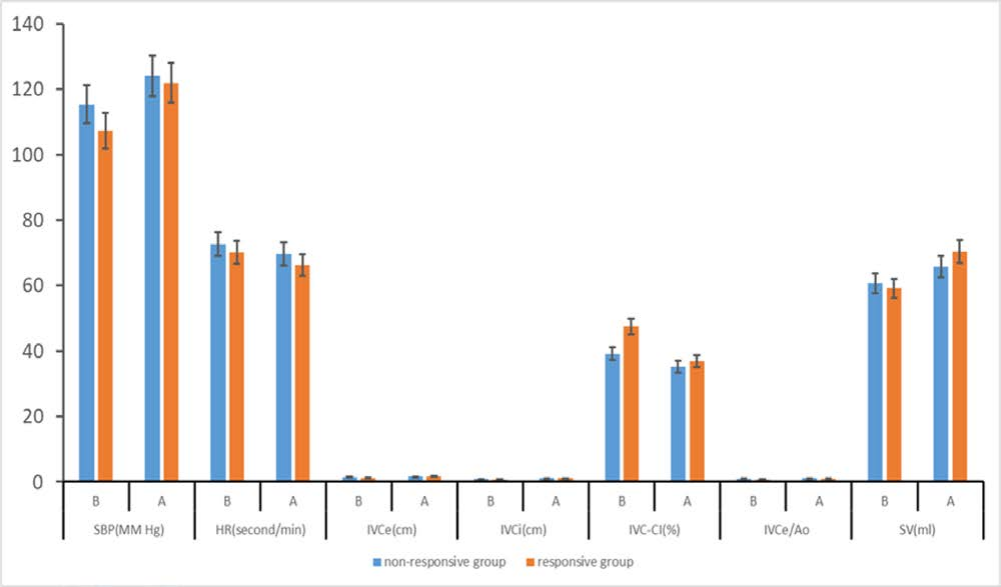
Comparison of relevant parameters before and after volume load test between 2 groups of patients (*x̅*±s).

Before the volume loading test, there were statistically significant differences between the 2 groups in terms of SBP, IVCe, IVCi, IVC-CI, and IVCe/Ao (*P* < .05). After the volume loading test, these parameters in the responsive group became similar to those in the nonresponsive group, and the differences were not statistically significant (*P* > .05). There was no significant difference in SV between the 2 groups before the volume loading test (*P* > .05), but after the test, the SV in the responsive group was significantly higher than in the nonresponsive group (*P* < .05). See Figure 1.

### 3.3. Comparison of the incidence of hypotension between the 2 groups

Out of 82 patients, 25 (30.49%) experienced hypotension after spinal anesthesia. The incidence of hypotension was 51.28% (20/39) in the volume-responsive group and 11.63% (5/43) in the volume-nonresponsive group, with a statistically significant difference (*χ*^2^ = 15.174, *P* < .01).

### 3.4. Comparison of inferior vena cava parameters for evaluating volume response after subarachnoid anesthesia

**Figure 2.**
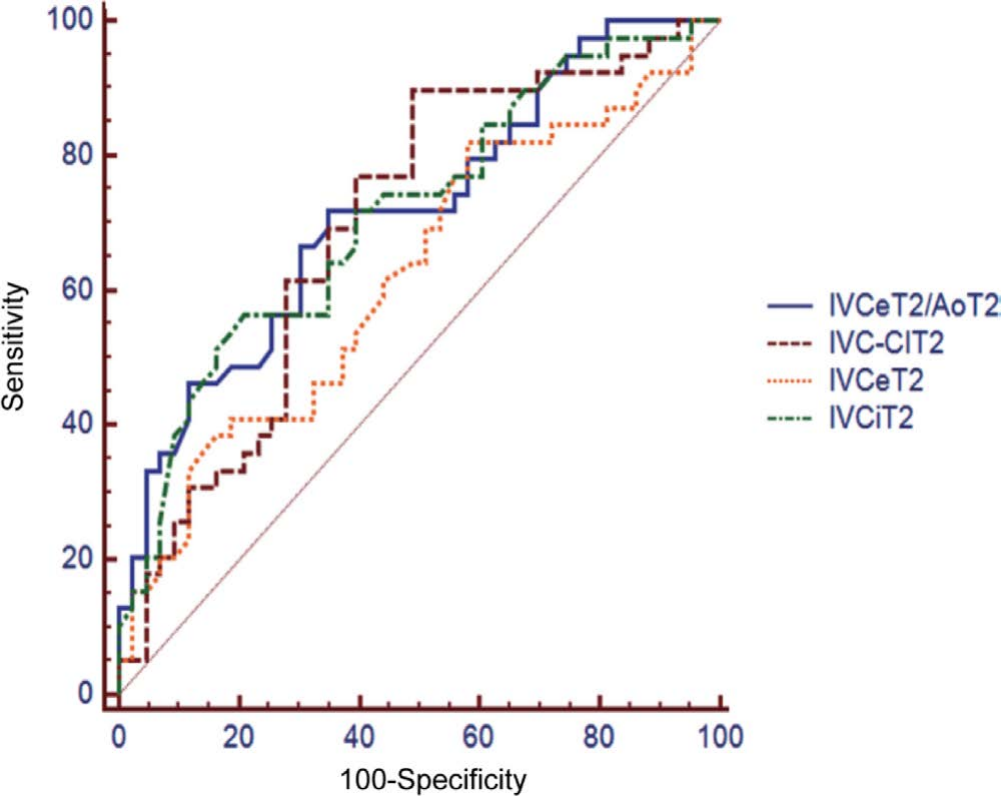
ROC curve for predicting volume responsiveness of inferior vena cava parameters before capacity load test. ROC = receiver operating characteristic.

The ΔSV in the responsive and nonresponsive groups of the volume load test were (19.58 ± 5.97)% and (8.13 ± 3.70)%, respectively, with a statistically significant difference (*P* < .05). ROC curves were plotted for IVCe, IVCi, IVC-CI, and IVCe/Ao to evaluate their reliability in predicting responsiveness to volume therapy after spinal anesthesia. The AUCs were 0.62, 0.71, 0.70, and 0.72, respectively. Among them, IVCi, IVC-CI, and IVCe/Ao could predict responsiveness to volume therapy after anesthesia (*P* < .05). Refer to Table 2 and Figure 2.

**Table 2:** ROC analysis results for predicting inferior vena cava parameters ΔSV = 15%.

Variable	Boundary value	Sensitivity (%)	Specificity (%)	ROC area	Standard error	*P*	95% CI
IVCeT1	1.56	82.1	41.9	0.62	0.06	>.05	0.51–0.73
IVCiT1	0.60	56.4	79.1	0.71	0.06	<.05	0.60–0.81
IVC-CIT1	36.71	89.7	51.2	0.70	0.06	<.05	0.59–0.79
IVCeT1/AoT1	0.84	71.8	65.1	0.72	0.06	<.05	0.61–0.81

*Note*: IVCeT1 is the IVCe at the end of 10 min after spinal anesthesia; IVCiT1 is the IVCi at the end of 10 min after anesthesia; IVC-CI T1 is the IVC-CI at the end of 10 min after anesthesia; IVCeT1/AoT1 is the IVCe/Ao at the end of 10 min after anesthesia.

ROC = receiver operating characteristic.

### 3.5. Comparison of inferior vena cava parameters in predicting persistent hypotension after spinal anesthesia

**Figure 3.**
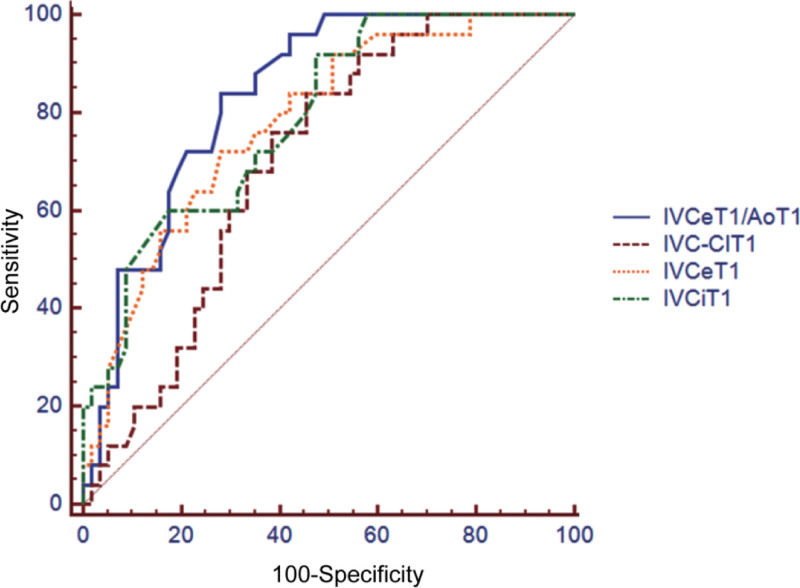
ROC curve prediction of hypotension after subarachnoid anesthesia with different parameters of inferior vena cava. ROC = receiver operating characteristic.

Subsequently, we analyzed the prognosis of hypotension in the volume response group. In the volume response group, 20 patients had hypotension after anesthesia, of which 9 had persistent hypotension and 11 had good improvement after treatment. We plotted ROC curves for IVCe, IVCi, IVC-CI, and IVCe/Ao to assess their reliability in predicting persistent hypotension after spinal anesthesia. The area under ROC curve (AUC) was 0.78, 0.79, 0.70, and 0.84, respectively, and the differences were statistically significant (*P* < .05). See Table 3 and Figure 3.

**Table 3:** ROC analysis results of predicting hypotension after subarachnoid anesthesia using inferior vena cava parameters.

Variable	Boundary value	Sensitivity (%)	Specificity (%)	ROC area	Standard error	*P*	95% CI
IVCeT2	1.41	72.0	71.9	0.78	0.05	<.05	0.68–0.86
IVCiT2	0.88	92.0	52.6	0.79	0.05	<.05	0.69–0.87
IVC-CIT2	42.97	84.0	54.4	0.70	0.06	<.05	0.59–0.80
IVCeT2/AoT2	0.87	84.0	71.9	0.84	0.04	<.05	0.74–0.91

*Note*: IVCeT2 is the IVCe at the end of 5 min of entry; IVCiT2 is the IVCi at the end of 5 min of entry; IVC-CIT2 is the IVC-CI at the end of 5 min of entry; IVCeT2/AoT2 is the IVCe/Ao at the end of 5 min of entry.

ROC = receiver operating characteristic.

### 3.6. Other findings

The highest level of anesthesia in both groups ranged from T12 to T6, with the median level at T10, showing no statistically significant difference (*Z* = 0.431, *P* = .667). There was a statistically significant difference in the number of patients who received norepinephrine within 30 minutes after anesthesia between the 2 groups (*χ*² = 10.802, *P* < .01), with 2 patients (5%) in the unreactive group and 14 patients (56%) in the reactive group. One patient received atropine treatment due to bradycardia. Within 30 minutes after anesthesia, 1 patient in each group experienced chills; however, no patients reported dizziness, nausea, or vomiting.

## 4. Discussion

Spinal anesthesia-induced hypotension is the most common complication, and clinical practice often involves pre-anesthesia preload or rapid infusion postanesthesia to reduce its incidence.^[15–17]^ However, inappropriate volume therapy can lead to fluid overload and related complications. Therefore, accurately assessing a patient’s volume responsiveness is crucial for guiding appropriate fluid therapy. Additionally, clinical studies have found that, despite good volume responsiveness, hypotension may still not improve, resulting in persistent hypotension.^[18–20]^ This study aims to explore the value of ultrasound-measured IVC parameters (IVCe, IVCi, IVC-CI, and IVCe/Ao) in assessing volume responsiveness and predicting persistent hypotension following spinal anesthesia.

### 4.1. Clinical significance of volume responsiveness group and nonresponse group

Patients in the volume responsiveness group showed a significant increase in stroke volume following fluid therapy, indicating better fluid tolerance and cardiac preload reserve in these patients. In contrast, the nonresponsive group may require additional measures to maintain hemodynamic stability. This finding emphasizes the importance of individualized fluid management, avoiding the risks associated with fluid overload while ensuring effective treatment for hypotensive patients.

### 4.2. Inferior vena cava parameters reflecting volume responsiveness

Before the volume loading test, there were statistically significant differences in systolic blood pressure, IVCe, IVCi, IVC-CI, and IVCe/Ao between the 2 groups (*P* < .05). After the volume loading test, the parameters in the responsive and nonresponsive groups were largely consistent, with no statistically significant differences (*P* > .05). This suggests that IVC parameters have good potential for predicting volume responsiveness. Furthermore, the study results showed that both IVCe and IVCi decreased significantly after spinal anesthesia, while IVC-CI increased significantly, which is consistent with vasodilation and reduced venous return caused by sympathetic blockade due to local anesthetics. After fluid loading, IVCe and IVCi increased, and IVC-CI decreased, indicating that IVC parameters can effectively reflect changes in volume status. In addition, changes in the IVCe/Ao ratio further corrected for individual differences, improving assessment accuracy. Moreover, the AUCs of the ROC curves for IVCe, IVCi, IVC-CI, and IVCe/Ao were 0.62, 0.71, 0.70, and 0.72, respectively. Among these, IVCi, IVC-CI, and IVCe/Ao could predict responsiveness to volume therapy after anesthesia (*P* < .05). These findings indicate that IVCe, IVCi, IVC-CI, and IVCe/Ao not only reflect changes in volume status before and after spinal anesthesia but also predict volume responsiveness.^[21,22]^

### 4.3. Inferior vena cava parameters predicting persistent hypotension

To explore the impact of IVC parameters on persistent hypotension, we divided hypotensive patients with volume responsiveness into those with persistent hypotension and those without, and conducted predictive estimation. Among the 20 hypotensive patients in the volume-responsive group, 9 experienced persistent hypotension, while 11 had a favorable treatment outcome. This finding confirms that hypotension caused by anesthesia cannot be solely explained and assessed by volume responsiveness.^[23]^ Therefore, we attempted to evaluate using postanesthesia IVC parameters. This study found that postoperative measurements of IVCe, IVCi, IVC-CI, and IVCe/Ao could predict persistent hypotension following spinal anesthesia, with the predictive ability of IVCeT2/AoT2 being particularly significant (optimal threshold of 42.97%). These findings indicate that these postoperative IVC parameters can effectively predict whether fluid therapy can improve hypotension, that is, predict the prognosis of patients’ response to fluid therapy.

### 4.4. Comparison of IVC parameters with traditional volume assessment methods

Traditional static preload indicators, such as central venous pressure and pulmonary artery wedge pressure, have limited effectiveness in predicting fluid responsiveness.^[24,25]^ In contrast, the IVC parameters used in this study, measured noninvasively via ultrasound, provide a more dynamic and individualized assessment that is also simple and fast. Additionally, measuring the abdominal aorta diameter corrects for the influence of body size on the IVC diameter, with the IVCe/Ao ratio as a composite indicator showing superior predictive accuracy compared to individual IVC parameters.^[26,27]^ This finding is consistent with the study by Kosiak et al, who explored the use of the IVCe/Ao ratio for assessing volume status and found it to be more reliable.^[8]^

### 4.5. Limitations of the study

This study has several limitations. First, SV was measured using echocardiography rather than invasive blood pressure monitoring, which may affect the accuracy and real-time nature of the measurements. Second, the time intervals between measurements of blood pressure and IVC parameters were relatively long, which might have failed to capture dynamic changes in real time, potentially missing some transient hemodynamic fluctuations. Future research should shorten monitoring intervals and increase measurement frequency to more comprehensively reflect patients’ hemodynamic status. Additionally, this study did not compare IVC parameters with traditional volume assessment indicators.

### 4.6. Clinical significance and future perspectives

In summary, this study demonstrates that IVC-CI, IVCe, IVCi, and IVCe/Ao effectively reflect volume responsiveness and predict persistent hypotension at different time points. In particular, the IVCe/Ao ratio, as a composite indicator, showed higher predictive accuracy compared to individual IVC parameters. This provides a reliable, noninvasive tool for guiding perioperative fluid management, helping to maintain hemodynamic stability, ensure patient safety, and promote rapid postoperative recovery. Future research should aim to further optimize monitoring methods, validate these parameters in broader clinical settings, and compare them with traditional assessment methods to enhance the scientific basis and effectiveness of volume management strategies.

## Author contributions

**Conceptualization:** Tao Yang, Chunyan Huang, Yulin Chen, Xuemin Lei.

**Data curation:** Tao Yang, Chunyan Huang, Yulin Chen, Xuemin Lei.

**Formal analysis:** Tao Yang, Chunyan Huang, Xuemin Lei.

**Investigation:** Tao Yang, Chunyan Huang, Yulin Chen.

**Methodology:** Tao Yang, Chunyan Huang, Yulin Chen, Xuemin Lei.

**Writing – original draft:** Tao Yang, Chunyan Huang.

**Writing – review & editing:** Tao Yang, Chunyan Huang.

**Supervision:** Xuemin Lei.

**Validation:** Xuemin Lei.
